# Quality of Life in Iranian Chemical Warfare Veteran's

**DOI:** 10.5812/ircmj.5323

**Published:** 2014-05-05

**Authors:** Abbas Ebadi, Tayeb Moradian, Mohsen Mollahadi, Yaser Saeed, Ali Akbar Refahi

**Affiliations:** 1 Behavioral Sciences Research Center (BSRC), Nursing Faculty of Baqiyatallah University of Medical Sciences, Tehran, IR Iran; 2 Department of Medical Surgical, Faculty of Nursing, Baqiyatallah University of Medical Sciences, Tehran, IR Iran; 3 Department of Pediatric, Faculty of Nursing, Baqiyatallah University of Medical Sciences, Tehran, IR Iran; 4 Department of Critical Care, Faculty of Nursing, Baqiyatallah University of Medical Sciences, Tehran, IR Iran

**Keywords:** Quality of Life, Chemical Warfare, Veteran, Chronic Disease

## Abstract

**Background:**

Mustard gas has different effects on different body systems such as respiratory tract, blood, gastrointestinal, skin, eye, endocrine and peripheral nervous system.

**Objectives:**

The purpose of this study was to determine the quality of life in chemical warfare veterans due to sulfur mustard exposure.

**Patients and Methods:**

In a cross-sectional and analytic study, 242 patients who had a chemical injury during the Iran-Iraq war (1980-1983) and their lung damage was proven were investigated in our study. The quality of life was measured in these patients using an extensively validated Iranian version of SF-36.

**Results:**

The mean age of veterans was 44.12 ± 4.9 ranging from 22 to 62 years. Our results showed that chemical warfare had a decreased quality of life in all subscales of the SF-36. The lowest scores in SF-36 subscales were related to role physical and general health. The data also showed a significant relationship between the number of organs involved and the quality of life in these patients (P < 0.001, r = − 0.33). So that the patients who had more than three organs involved had lower quality of life. 95.4% of our participants experienced another complication with respiratory complication and the ophthalmologic complications were the most frequent accompanying condition.

**Conclusions:**

The results imply that chemical warfare survivors suffering from late complications have a low health related quality of life.

## 1. Background

The mustard gas is the most used chemical agent in Iran-Iraq war against Iranian and has many long term complications (1, 2). According to the available reports more than 100,000 people are suffering from chemical injuries due to sulfur mustard (3). Different effects of mustard gas on different body systems such as respiratory tract, blood, gastrointestinal, skin, eye, endocrine, peripheral nervous system, genetic alterations and carcinogenesis have been reported (2-7). These complications are chronic and progressive and continuously affect the quality of life in these patients (8). This gas has a long-term irreversible adverse effect on the respiratory system and cause disability, acute and chronic adverse effects (9, 10). More than 80% of Iranian chemical victims suffer from cough, respiratory discharges and dyspnea (11). These symptoms affect different aspects of patient’s life, such as family and social role function and finally reduce patient’s quality of life. Chemical complications in these patients are progressive and over time they will increase in amount and severity (8). Recent studies showed that ophthalmologic complication in these patients is correlated with reduced psychological health status (12).

## 2. Objectives

Since the exposure to sulfur mustard is considered as a chronic status, cure remains elusive, and death is distant. In these situations, the aim of treatments and care is to increase the longevity and enhance the patient’s ability to achieve an appropriate level of quality of life (13, 14). Researches has shown that pulmonary disease has a significant effect on activities of daily living (15) and these patients even in the middle stages of the disease have a low quality of life (16). Chronic diseases cause a lot of physical and mental stress on patient and his family, and create lifelong changes in roles, lifestyle, and lead to frequent hospitalizations, financial problems and decreased social interaction between family members (17, 18). Now-a-days for determining health needs and improving the quality of health care, the quality of life is measured. The current study was conducted to evaluate the quality of life in chemical warfare survivors suffering from late complications due to sulfur mustard exposure.

## 3. Patients and Methods

In a cross-sectional and analytic study the quality of life in Iranian chemical veterans was assessed. Injured survivors of the Iran-Iraq war who are referred to Veterans and Martyrs Affair Foundation (VMAF) are given a severity index (disability rate) in the VMAF database, based on their clinical problems and severity of the injury or injuries. This database keeps all the victims information consisting demographic data and medical history. Using the convenient sampling 242 patients who had a chemical injury during the Iran-Iraq war (1980-1983) and their lung damage was proven were investigated in our study. All patients had exposure to sulfur mustard during this period. Data for age, frequency of exposure to sulfur mustard, history of hospitalization, level of education, percentage of disability and number of injured organs was extracted from VAMF database. Diagnosis of lung damage in these patients was previously given by the medical committee, and the diagnostic tests were proven by a lung specialist. These patients were evaluated during the referral to the Baqiyatallah Hospital Clinic. Quality of life was measured using an extensively validated Iranian version of SF-36 (19). The SF-36 is a generic tool that can be used for the general population and different patients groups. This questionnaire is widely used and consists of 36 items. It also contains 36 items divided into eight domains: Physical Functioning (PF), Role-Physical (RP), Bodily Pain (BP), General Health (GH), Vitality (VT), Social Functioning (SF), Role-Emotional (RE) and Mental Health (MH). It also provides two summary scales: Physical Component Summary (PCS) and Mental Component Summary (MCS). Scores for each subtitle range from 0 to 100, which 100 representing the best health related quality of life and 0 representing the worst (20). The validity and reliability of Iranian version of this questionnaire is previously assessed by Ali Montazeri (19). The ethics committee affiliated with the Janbazan Medical & Engineering Research Center (JMERC) approved the study. After explaining the study object to the participants, they were informed that participation in the study is voluntary and that they could refuse to participate in the study without being penalized. Last, written informed consent according to the provisions of the Declaration of Helsinki was obtained from the participants who agreed to participate in the study. The data were analyzed using SPSS software version 17. In addition to descriptive statistics, our data was analyzed by independent t test, one-way ANOVA and Pearson correlation coefficient. A P value less than 0.05 was considered as statistically significant.

## 4. Results

This study was conducted to assess the quality of life in Iranian chemical warfare suffering from late complications due to sulfur mustard exposure. The study was performed during December 2009 to June 2010. All participants were male, and majority (99.6%) of them was married. The mean age was 44.12 ± 4.91 ranging from 25 to 62 years. Other demographic data are shown in Table 1. Our study participant quality of life was assessed, and our results showed that chemical warfare had a decreased quality of life in all subscales of the SF-36 (Table 2). The quality of life in Physical Component Summary (PCS) subscale was 32.31 ± 16.8 and in Mental Component Summary (MCS) was 38.36 ± 19.33. This results shows that the quality of life in both subscales was low. There was no correlation between the percentage of disability and the quality of life (P = 0.80, r = 0.01), but the association between the number of organs involved and the quality of life was significant (P < 0.001), so that patients who had more than three organs involved had lower quality of life. Also, we tested the association between level of education and quality of life. Patients who had academic education had a better quality of life compared to those who were low educated (28.16 ± 3.11 vs. 24.11 ± 2.27, P < 0.001).

In this study, we assessed the accompanying complication with pulmonary complications. In this study, 95.4% of our participants experienced another complication. Ophthalmologic complication with an incidence of 82.33% and skin and psychiatric complication with an incidence of 76.85% were the most common accompanying complications with lung injury. 71.1% of participants had more than two complications.

**Table 1.** Demographic Characteristics of Iranian Chemical Warfare Suffering From Pulmonary Complications

Characteristics	Values
**Age, y**	44.12 ± 4.9
**Disability percent**	28.71 ± 18.83
**Marital (married)**	241 (99.6)
**Education (academic)**	97 (40.1)
**Organs involved ( more than 3)**	172 (71.1)
**Time from exposure, y**	23.05 ± 1.48
**Initiating the symptoms, y**	16.98 ± 6.95

**Table 2.** SF-36 Scores in Iranian Chemical Warfare Suffering From Pulmonary Complications^a^

Subscale	Mean ± SD	95% Confidence Interval	
-	-	Lower Bound	Upper Bound
**Physical functioning**	43.24 ± 22.75	40.30	46.10
**Role physical**	20.97 ± 25.70	17.71	24.22
**Bodily pain**	35.23 ± 19.33	32.78	37.68
**General health**	29.81 ± 18.57	27.64	32.26
**Vitality**	35.10 ± 20.58	32.49	37.70
**Social functioning**	44.73 ± 23.29	41.73	47.68
**Role emotional**	30.85 ± 33.44	26.61	35.08
**Mental health**	42.77 ± 21.84	40.01	45.54
**PCS** ^**a**^	32.31 ± 16.08	35.91	40.81
**MCS** ^**a**^	38.36 ± 19.33	32.27	37.40

^a^ Abbreviations: PCS, physical component summary; MCS, mental component summary.

## 5. Discussion

The results of the present study show that the chemical warfare with late complications had low quality of life. This result is consistence with other studies (21-25). And some studies report lower quality of life in survivors of chemical weapons compared with other war survivors not exposed to chemical weapons (12, 26). Late adverse effects of chemical weapons can cause some limitations in physical, psychological and social aspects of one's life and diminish the quality of life. The findings revealed that participants particularly scored lower on the role physical and general health subscales. These findings are similar to Mousavi study (25). Overall Mental Component Summary (MCS) sub-score was higher than Physical Component Summary (PCS). Regarding the chronic adverse effects of mustard gas and fear of subsequent complications and prolonged stress in the lives of these veterans, mental health disorders in these patients is not unexpected. A possibility that most of the Iranian war victims were volunteer veterans and civilians and hence copped better with their conditions, could be considered. Also, must of them enjoy from having a supportive family environment. Ebadi categorize adaptation sources in these patients in 4 main categories including religious factors, patriotism, social support and attitude toward the disease. The most important adaptation source in his study was religious factor (27). It seems that programs should be designed to strengthen these sources of compatibility. In this study, there was no significant correlation between disease severity and quality of life scores, but some studies reported that this correlation is high (23, 24), and some others mentioned no correlation between disease severity and quality of life (25). For example, Attaran reported a significant correlation between disease severity and quality of life (r = 0.51). We also assessed the relation between the number of organs involved and quality of life. Findings revealed that there is a significant association between the number of diseased organs and quality of life. So that patients who had more than three organs involved had lower quality of life. It seems that number of organs involved is more important than disability percent. So health care system should have a more detail consideration on patients with more organs involved. Patients who had higher education had a better quality of life. Patients who had academic education had a better quality of life compared to those without academic education. Our result is consistence with other studies (25). This can be interpreted in two categories. The first implies the role of academic education in better quality of life, and the second might be due to the fact that the SF-36 is highly dependent on education. Simultaneous involvement of different organs consist of eye, skin, lung, psychological disorders cause diminished quality of life. So in the case of planning for increasing the quality of life in these chronic patients, in the same extent that we consider pulmonary complications we should pay attention to other accompanying disease. And it is necessary that professionals from different disciplines cooperate to improve veterans’ health. Studies have also shown that patients participating in the war who had continuous following treatment had a better health related quality of life (28). Exercise also can be another resource of compatibility in these patients. Mousavi mentioned a better quality of life for chemical warfare involved in sport programs (25). The results imply that chemical warfare survivors suffering from late complications have a low health related quality of life. It is recommended that health policymakers consider problems of these veterans who volunteered and have participated in the war. Further researches are needed to measure the effect of different interventions on quality of life in these patients. In addition, awareness of the copping resources and enhancing them in these patients can cause a better copping with these conditions and improvement in health promotion.
